# Bowel and related complications after cardiac surgery

**DOI:** 10.1186/cc14455

**Published:** 2015-03-16

**Authors:** CK Kerneis, AL Lafarge, LL Larnier, F Scalbert, AB Brusset, PE Estagnasie, PS Squara

**Affiliations:** 1 Clinique Ambroise Paré, Neuilly-sur-Seine, France

## Introduction

Postoperative ileus appears to be underestimated after cardiac surgery. We conducted this study to analyse the incidence, risk factors and outcomes of postoperative ileus.

## Methods

In this single-centre observational study we prospectively enrolled all patients undergoing elective cardiac surgery. The primary output was the time to faeces (TTFE) as representing the postoperative ileus. Secondary outputs were the occurrence of ischaemic colitis and pneumonia. Quantitative variables were compared by ANOVA or Wilcoxon tests, qualitative variables by chi-square tests. Multivariate analyses were performed by logistic regression, *P *< 0.1 for inputs *P *< 0.05 for outputs.

## Results

**Figure 1:**
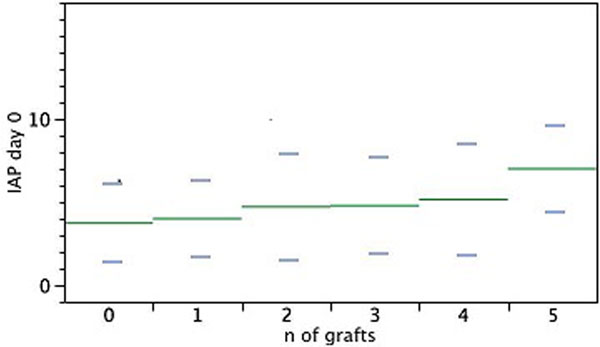


We included 349 patients: age 67.5 ± 10.8 years, M/F sex ratio 252/97, preoperative left ventricle ejection fraction 58.8 ± 10.6%, bypass/valve ratio 234/154, number of grafts 2.7 ± 0.9, mammal arteries 1.8 ± 0.5. In univariate analyses, bypasses received more anaesthetic drugs (*P *< 0.01), had shorter extracorporeal circulation duration, 67 ± 27 versus 75 ± 24 minutes (*P *< 0.01), and received less blood products (*P *< 0.0001). Bypasses had lower postoperative levels of troponin (3.9 ± 7.6 vs. 8.1 ± 21 pg/ml, *P *< 0.01) and LDH (330 ± 162 vs. 420 ± 175 pg/ ml). In contrast, the intra-abdominal pressure (IAP) was higher and related to the number of grafts at day 0 (Figure 1) and day 1 (*P *= 0.01 and 0.02 respectively), and to the number of mammal grafts at day 0 and day 1 (*P *= 0.01 and 0.04 respectively). The TTFE was longer but did not reach significance (*P *= 0.13) as well as the occurrence of abdominal ischaemia (*P *= 0.22). The occurrence of pneumonia was higher (*P *= 0.01). In multivariate analysis, the IAP at day 0 and day 1 was related to propofol quantities only. The predictors of pneumonia were: duration of mechanical ventilation, peak lactate in the postoperative 24 hours, and coronary bypass: OR = 163, 2.6, and 4.2 respectively.

## Conclusion

The number of coronary grafts and of mammal artery used in cardiac surgery is associated with higher IAP and higher risk of pneumonia. However, whether this is due to direct bowel ischaemia or longer anaesthesia remains to be studied in larger trials.

